# Camrelizumab-induced immune-related toxic epidermal necrolysis in lung adenocarcinoma: a case report and literature review

**DOI:** 10.3389/fonc.2024.1417936

**Published:** 2025-01-13

**Authors:** Man Sun, Huan Zhou, Dan Zang, Chen-Guang Liu, Jun Chen

**Affiliations:** Department of Oncology, The Second Hospital of Dalian Medical University, Dalian, Liaoning, China

**Keywords:** lung adenocarcinoma, camrelizumab, immune-related adverse events, toxic epidermal necrolysis, drug adverse reactions

## Abstract

Toxic epidermal necrolysis (TEN) is a rare and serious skin reaction. This study reports a case of a patient with lung adenocarcinoma (LUAD) who developed severe TEN after 8 days of treatment with Camrelizumab monotherapy. The patient’s condition was effectively relieved with high-dose corticosteroids and intravenous immunoglobulin therapy. The diagnosis and treatment of immune-related TEN are challenging. This is a rare and severe case of TEN associated with the use of Camrelizumab. Additionally, we provide an in-depth understanding of immune-related TEN, summarizing its characteristics and treatment progress through a literature review, aiming to provide reference for the clinical safe application of immune checkpoint inhibitors (ICIs).

## 1 Introduction

According to the 2020 global cancer statistics, the incidence of lung cancer is as high as 11.4%, ranking second among newly diagnosed cancer cases (1). Non-small cell lung cancer (NSCLC) accounts for approximately 80-85% of lung cancer cases, with LUAD being the most common histological type. In recent years, the emergence of monoclonal antibodies targeting programmed cell death 1 (PD-1), programmed cell death ligand 1 (PD-L1), and cytotoxic T-lymphocyte-associated protein 4 (CTLA-4) has changed the treatment landscape for locally advanced or metastatic NSCLC without oncogenic driver gene mutations (2, 3). Compared to monotherapy with chemotherapy, ICIs targeting the PD-1 axis have demonstrated significantly improved patient survival rates in first-line or second-line treatment for advanced NSCLC, whether used alone or in combination with chemotherapy (4).

Camrelizumab is a novel humanized anti-PD-1 IgG4 monoclonal antibody developed independently in China. It was approved by the China Food and Drug Administration (CFDA) in June 2020 for first-line treatment of advanced non-squamous NSCLC patients in China who are ineligible for chemotherapy and lack epidermal growth factor receptor (EGFR) and anaplastic lymphoma kinase (ALK) mutations (5). Studies by Zhou et al. have shown that treatment-naive advanced NSCLC patients receiving camrelizumab in combination with chemotherapy demonstrated significant improvements in progression-free survival (PFS) and overall survival (OS) compared to those receiving chemotherapy alone (6, 7). Camrelizumab has demonstrated durable clinical efficacy in some NSCLC patients, albeit accompanied by various immune-related adverse events (irAEs), including neutropenia, leukopenia, anemia, and rash-like cutaneous adverse events (RCCEP), ranging in severity from mild to life-threatening (7–9). Skin irAEs are typically the most common irAEs and occur early.

This article reports a case of immune-related TEN in a patient with LUAD treated with camrelizumab, and comprehensively explores its clinical characteristics and management strategies through a literature review.

## 2 Case presentation

The patient, a 58-year-old female weighing 55 kg with a Body Mass Index (BMI) of 21.48, was diagnosed with stage IV left LUAD in August 2023. The patient had a history of well-controlled hypertension, managed with self-administered nifedipine, and was not on any other medications. The patient and her family have no history of immune-related diseases. Immunohistochemistry results showed CK8/18(+), NapsinA(+), TTF-1(+), EA(+), CR (-), MC(-), WT-1(-), CK5/6(-), P40(-), and p63(-).PD-L1 testing suggested a potentially high benefit from PD-1/PD-L1 class ICIs. Despite no detectable mutations for targeted therapy, the patient self-administered gefitinib from August 24 to November 15, 2023. Subsequently, upon further examination at our hospital, it was confirmed that the patient was not eligible for surgery. After excluding contraindications, the patient received single-agent camrelizumab injection at a dose of 200 mg intravenously on November 22, 2023. Due to the patient and family’s refusal of systemic chemotherapy, opting only for localized infusion, the patient received a 30 mg intrapleural cisplatin infusion on December 1, 2023. Although the standard treatment would include combined chemotherapy and immunotherapy, systemic chemotherapy was not administered per their decision. The current treatment approach is primarily focused on symptomatic relief, particularly through thoracentesis for pleural effusion management.

On December 1, 2023, the patient developed a fever, reaching a maximum of 38.2°C, accompanied by generalized body pain. She self-administered ibuprofen orally to alleviate fever and pain symptoms. Three days later, the patient developed scattered red rashes throughout her body, starting from the back and gradually spreading to the upper abdomen, upper limbs, and lower limbs, merging into patches, accompanied by pain and itching. The rash surface developed blisters and ruptures, and ulceration appeared in the oral mucosa. The patient attempted to alleviate the rash by taking oral antiallergic medications, but there was no improvement. On December 9, she was admitted to the hospital for treatment.

**Figure 1:**
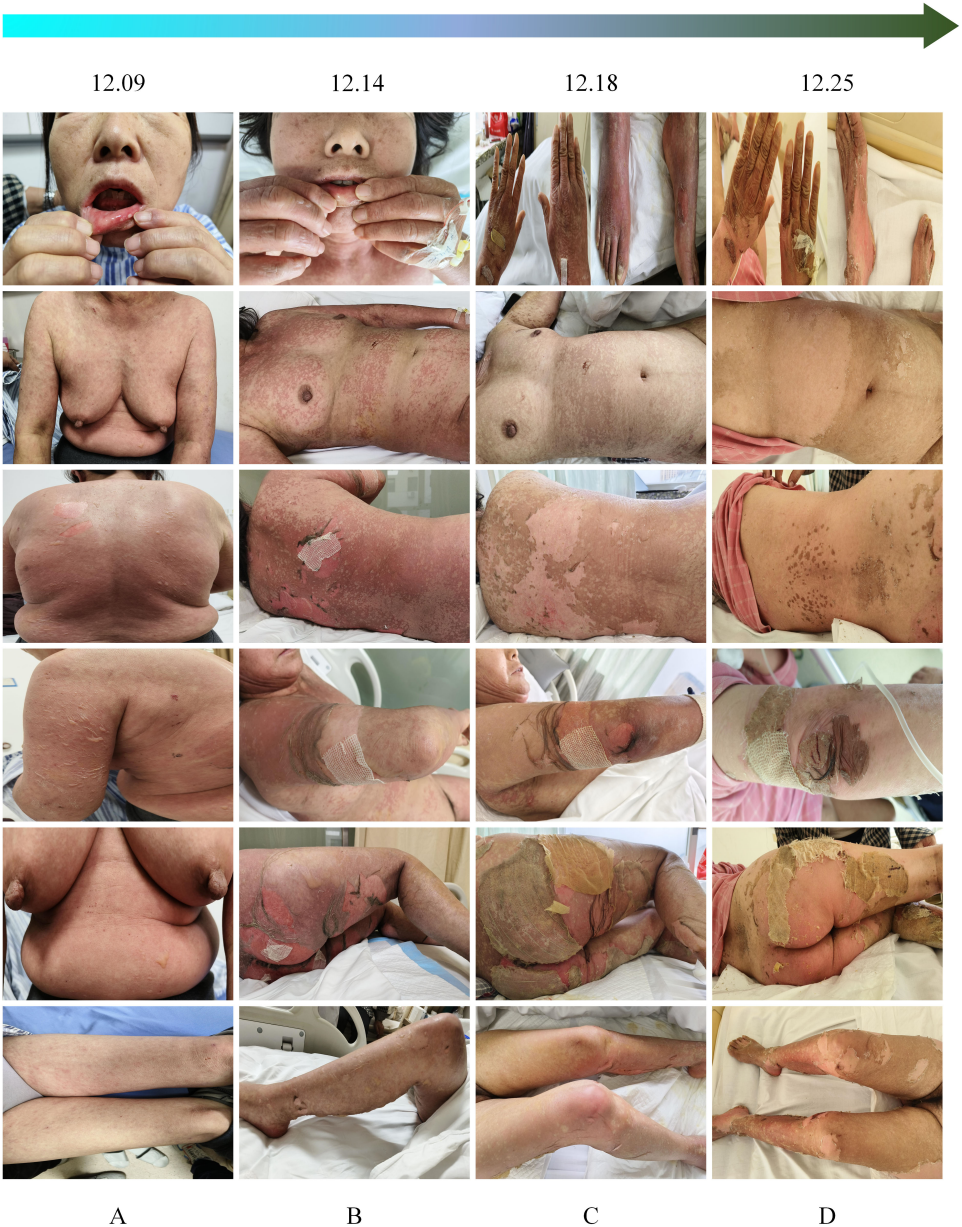
Progression of the dermatological condition over time. **(A)** December 9: Initial presentation of lesions. **(B)** December 14: Progression with increased scaling and erythema. **(C)** December 18: Further deterioration, with prominent desquamation and ulceration. **(D)** December 25: Partial healing with residual hyperpigmentation and scarring.

After admission, relevant examinations were conducted, and the physical examination revealed a positive Nikolsky sign. Based on the patient’s medical history, medication history, and clinical manifestations, a preliminary diagnosis of immune-related TEN was made (**Figure 1A**). Treatment included intravenous administration of methylprednisolone 80 mg for 3 days (12.09-12.11).

**Figure 2:**
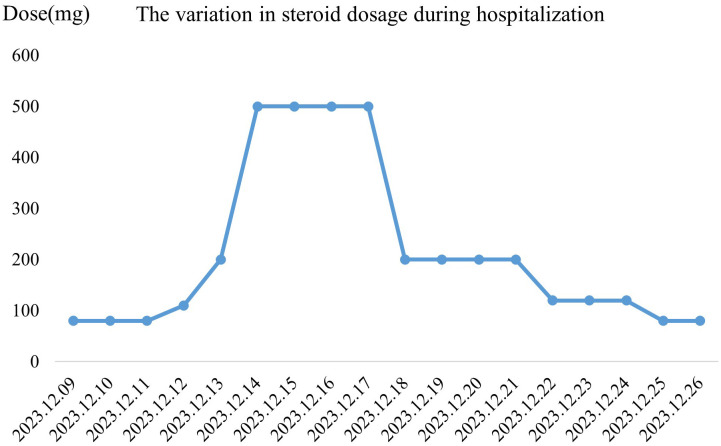
(We have corrected the image to reflect the appropriate y-axis label as ‘Dose’ and updated the title to ‘The variation in steroid dosage during hospitalization.) Variation in steroid dosage during hospitalization. The graph illustrates the changes in daily steroid dosage (mg) administered to the patient from December 9, 2023, to December 26, 2023. A rapid increase in dosage is observed from December 12 to December 14, reaching a peak on December 15–17, followed by a gradual tapering from December 18 onwards.

**Figure 3:**
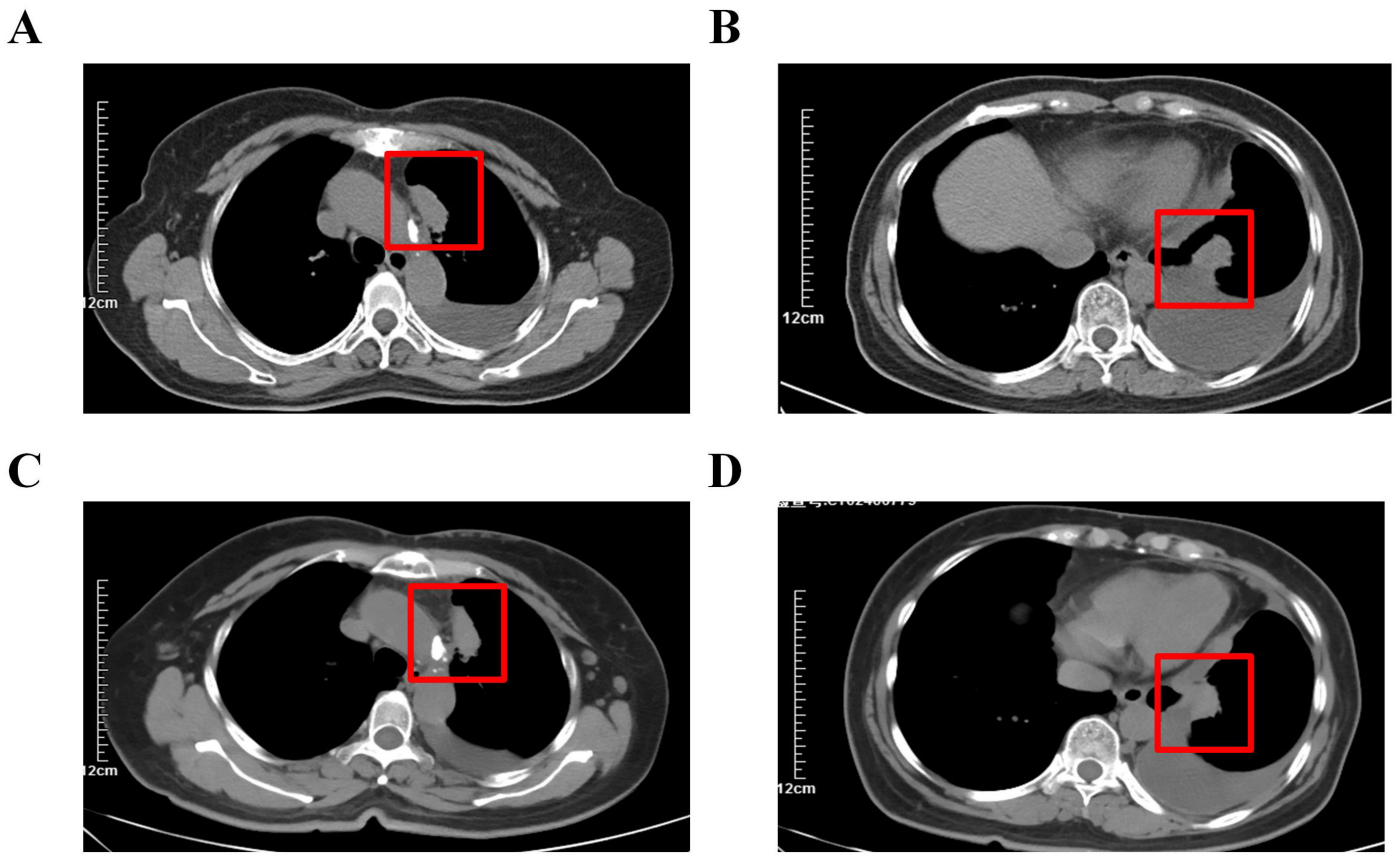
(We have updated the images to replace the previously submitted ones, ensuring that all patient information is properly concealed to protect privacy.) Changes in target lesion during treatment. **(A, B)** CT scan before treatment. **(C, D)** CT scan after 1 cycles of treatment. he red arrows mark the target lesions. The red box marks the target lesions.

**Figure 4:**
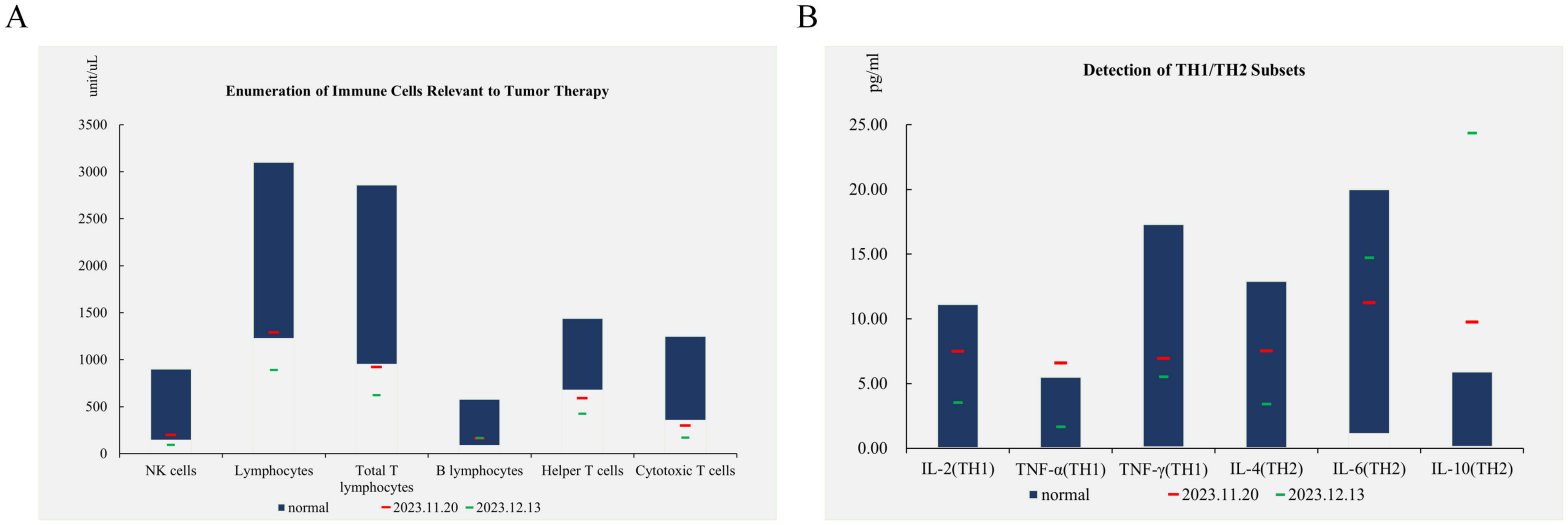
Analysis of immune cell counts and cytokine profiles related to tumor therapy. **(A)** Enumeration of immune cells relevant to tumor therapy, including NK cells, Lymphocytes, Total T lymphocytes, B lymphocytes, Helper T cells, and Cytotoxic T cells, comparing normal levels with patient levels on November 20, 2023, and December 13, 2023. **(B)** Detection of Th1/Th2 subsets, highlighting the levels of IL-2 (Th1), TNF-α (Th1), TNF-γ (Th1), IL-4 (Th2), IL-6 (Th2), and IL-10 (Th2) in normal controls and in the patient on November 20, 2023, and December 13, 2023.

Epidermal growth factor was topically applied to the skin detachment areas, while undamaged rash areas were treated with topical application of clobetasol, and oral ulcers were treated with Kangfu Xin liquid. Subsequently, the methylprednisolone dose was adjusted to 110 mg intravenously for 1 day (12.12), resulting in improvement of oral ulcers but an increase in the size of the generalized rash area, with local fluid leakage observed, rapid enlargement and fusion of newly developed blisters on both lower limbs. The rash exhibited patchy epidermal shedding and necrosis, involving nearly 30% of the body surface area. The rash was more severe in areas under pressure (e.g., buttocks, back, and posterior arms). As of December 13, 2023, the methylprednisolone dose was increased to 200 mg, and intravenous immunoglobulin 22.5 g was administered by drip for 8 days (12.13-12.20). The patient’s rash continued to worsen, involving nearly 70% of the body surface area. The methylprednisolone dose was adjusted to 500 mg intravenously for 4 days (12.14-12.17) (**Figure 1B**). By December 18, 2023, no new rashes were observed, blister leakage was reduced compared to before, some skin lesions began to scab, and the methylprednisolone dose was adjusted to 200 mg intravenously (12.18-12.21), with the addition of nystatin mouthwash for fungal infection prevention (**Figure 1C**). On December 22, 2023, steroid dosage was further reduced to 120 mg intravenously. By December 25, 2023, the rash leakage gradually decreased, pain significantly relieved, original blisters and erythema completely faded, and the skin began to scab, dry, and peel off, with no new rashes observed. The methylprednisolone dosage was reduced to 80 mg for two days (**Figure 1D**). After discharge, methylprednisolone was switched to oral prednisone 60 mg and gradually tapered over 30 days (**Figure 2**). During hospitalization, supportive treatments including anti-infection, anti-inflammatory, antiallergic, and symptomatic treatments were administered. Additionally, dynamic monitoring of lung-enhanced CT scans (**Figure 3**), blood routine, liver and kidney function, electrolytes, and other parameters revealed no significant abnormalities. Furthermore, detection of tumor treatment-related immune cells and Th1/Th2 subsets was performed (**Figure 4**). Currently, the patient is undergoing continuous treatment and monitoring, with no signs of rash recurrence.

## 3 Discussion

ICIs have made significant progress in cancer treatment; however, approximately 30% of patients may experience irAEs. A study showed that the overall incidence of any grade of irAEs in NSCLC patients was 30%, with a rate of 6% for severe irAEs, highlighting the balance between the potential risks and benefits of ICI therapy (10).

Chemotherapy can lead to excessive immune system activation, and in such cases, immune-related TEN may result from the combined effect of multiple drugs rather than a single agent. In this case report, the patient developed TEN within 8 days after receiving camrelizumab treatment. It is worth noting that immune-related skin adverse reactions typically occur 1 week to several months after immunotherapy, which is consistent with the typical temporal pattern observed in this case. Additionally, platinum-based injections can also lead to skin adverse reactions; however, in this patient, the treatment modality was intrathoracic instillation, and skin adverse reactions to cisplatin mainly occur after repeated treatment and intravenous administration (11). Considering these factors, we conclude that the patient’s skin reaction was mainly induced by the ICI, though the possibility of immune activation triggered by cisplatin cannot be entirely ruled out. According to the guidelines of the Chinese Society of Clinical Oncology (CSCO) on the management of ICIs-related toxicity and the Common Terminology Criteria for Adverse Events version 4.03 (CTCAE-4.03), the patient’s skin adverse reaction was assessed as grade 4, classified as a severe adverse event. Therefore, permanent discontinuation of ICI treatment is warranted.

The patient underwent contrast-enhanced CT scanning for cancer progression assessment on November 21st (prior to camrelizumab infusion). By December 12th, the left upper lobe mass increased from 32*15mm to 34*17mm, while the left lower lobe mass slightly decreased from 26*22mm to 23*20mm. Overall, there was no significant disease progression, and the therapeutic response was assessed as stable disease (SD). The patient did not continue with PD-1 antibody treatment. In a cohort study, 153 melanoma patients receiving ICIs as first-line treatment showed a higher 5-year overall survival rate in patients experiencing skin irAEs (12). Although previous studies have linked irAEs with better ICI efficacy, this patient, after one treatment cycle, showed no significant improvement (13, 14).

Among various irAEs, the skin is the most common site, occurring in over one-third of treated patients, manifesting as pruritus, maculopapular rash, lichenoid dermatitis, vitiligo-like pigmentary changes, and Stevens-Johnson syndrome/Toxic epidermal necrolysis(SJS/TEN) (15). SJS/TEN is a rare and life-threatening T-cell-mediated type IV hypersensitivity reaction, typically develops within 4 days to 4 weeks (16). SJS and TEN exhibit similar disease processes, with the main distinction being the extent of skin detachment (17). In SJS, epidermal detachment involves less than 10% of the body surface area (BSA), while in TEN, it exceeds 30% of the BSA. Clinical manifestations of ICIs-induced SJS/TEN resemble those induced by other drugs, presenting as erythema or atypical target lesions on the trunk, gradually evolving into confluent erythematous areas with central fading and separation of the epidermis and dermis (18–21). Mucosal involvement is observed in the majority of patients, with oral mucosa being the most commonly affected, occurring in up to 100% of cases. The mechanism of occurrence remains unclear, with various theories proposed, including the hapten/pro-hapten theory, alteration of peptide theory, drug immune receptor theory, and alteration of T cell receptor complex theory (22, 23). The mortality rate of SJS is as high as 10%, while that of TEN is as high as 50%, necessitating permanent discontinuation of immunotherapy once it occurs (24). Currently, the main treatment approach involves discontinuing the use of immunomodulatory drugs and employing high-dose corticosteroids, intravenous immunoglobulins, oral cyclosporine, and biologic TNF-α inhibitors (such as infliximab, etanercept), as well as methods such as blood dialysis, to reduce morbidity and mortality (16, 25).

A comprehensive PubMed search up to December 2022 identified 95 reported cases of immune checkpoint inhibitor (ICI)-induced SJS/TEN during immunotherapy (26). Additionally, a systematic review of camrelizumab-related TEN cases found only two instances in patients with esophageal squamous cell carcinoma and gallbladder cancer, treated either with monotherapy or combination therapy (27, 28). In summary, the occurrence of SJS/TEN induced by ICIs is generally rare, with camrelizumab-associated cases being even less common. Most patients experience symptom resolution following treatment with corticosteroids and intravenous immunoglobulin (IVIg).

The occurrence of immune-related SJS/TEN may involve changes in various immune cells. Studies have found that when ICIs activate certain drug-specific T lymphocytes, cytotoxic molecules and various pro-inflammatory cytokines are released, leading to inflammatory damage (29, 30). In addition to cytotoxic T cells, activated natural killer (NK) cells, T helper 17 cells (Th17), and antigen-presenting cells (APCs) also promote the release of pro-inflammatory cytokines, further exacerbating inflammatory damage and causing SJS/TEN. We conducted tumor treatment-related immune cell and Th1/Th2 subset detection in the patient, comparing the concentrations of 6 cytokines (IL-2, IL-4, IL-6, IL-10, TNF-α, and TNF-γ) and various immune cells in peripheral blood before and after treatment. After immunotherapy, the level of IL-10 significantly increased, but there was no significant imbalance between Th1 and Th2 in SJS/TEN (31). In addition, the counts of T cells, CTLs, and NK cells all significantly decreased. These findings suggest that specific cytokines and immune cells in peripheral blood may have potential as predictive biomarkers for SJS/TEN.

Cisplatin and methylprednisolone can significantly impact immune cells and cytokine profiles during treatment. Cisplatin upregulates MHC class I expression, modulates the immunosuppressive microenvironment, promotes the recruitment and proliferation of immune effector cells, and enhances their cytolytic activity (32). In contrast, glucocorticoids like methylprednisolone are used to counteract immune-mediated adverse effects by inhibiting dendritic cell maturation, downregulating MHC class II, co-stimulatory molecules, and pro-inflammatory cytokines, while promoting anti-inflammatory cytokines like IL-10. This suggests that the patient’s immune cell alterations may have been influenced by both ICI treatment and the concurrent use of cisplatin and methylprednisolone (33).

We have compiled a table reviewing the literature on rashes associated with Camrelizumab (27, 28, 34–38) (**Table 1**). While these studies overlap with ours, we provide a detailed description of clinical symptoms, treatment, outcomes, and immune response mechanisms, integrating serological and genomic analyses for a more comprehensive understanding of immune-related adverse events.

**Table 1:** Comparison of Camrelizumab-Related Rash Cases: A Review of Existing Literature.

No.	Author/year	Dose of Camrelizumab	Type of cancer	Regimen	Onset Timing	Skin Reaction Types
1	Yang et al., 2021 (28)	200mg	Gallbladder Carcinoma	Camrelizumab and Apatinib	3 days	Toxic Epidermal Necrolysis
2	Gao et al., 2022 (34)	*	Lung Cancer	Camrelizumab and Chemotherapy	<180 days	Depigmentation of the skin
3	Liu et al., 2022 (35)	200 mg	Esophageal Squamous Cell Carcinoma	Camrelizumab monotherapy	10 minutes after the second infusion	Generalized rash
4	Ouyang et al., 2023 (36)	*	Angioimmunoblastic T-cell lymphoma	Camrelizumab and Chidamide	180 days	Eosinophilic fasciitis
5	Peng et al., 2021 (27)	*	Esophageal Squamous Cell Carcinoma	Camrelizumab monotherapy	2 days	Toxic Epidermal Necrolysis
6	Wang et al., 2023 (37)	200 mg	Esophageal Squamous Cell Carcinoma	Camrelizumab monotherapy	1 year	Oral lichenoid reaction
7	Hu et al., 2024 (38)	200 mg	Squamous cell carcinoma of the floor of the mouth	Camrelizumab and Chemotherapy	10 minutes after the second infusion	Localized skin erythema

*Not explicitly mentioned in the article.

Clinical physicians should be vigilant about the potential occurrence of irAEs during immunotherapy, as early identification of these events contributes to better patient management and maximizes patient benefit whenever possible. A dermatology expert should be integral to the diagnosis and management of skin-related immune-related adverse events (irAEs) (39). Investigating the underlying mechanisms may be enhanced by incorporating microscopic biopsies of skin tissue and exudate alongside blood samples. Skin biopsies enable direct observation of cellular composition, immune cell infiltration, and cytokine responses, which are critical for diagnosing and distinguishing various irAEs. Exudate analysis can further reveal inflammatory mediators and cellular debris, providing a comprehensive view of the immune response. These insights are essential for developing targeted therapeutic strategies, such as selecting appropriate immunosuppressive treatments or identifying biomarkers for early detection and intervention (40). During treatment with Camrelizumab, patients should be closely monitored for skin reactions and seek medical attention promptly. The adoption of a multidisciplinary and interdisciplinary approach is crucial for facilitating early diagnosis and treatment of SJS/TEN, preventing permanent organ damage. In the future, further research is needed to elucidate the underlying mechanisms of immune-related adverse events, in order to extend the benefits of immunotherapy to more patients.

## 4 Conclusion

We present a case of a 58-year-old female with LUAD who developed immune-related TEN following treatment with camrelizumab. This case underscores the potential for severe cutaneous reactions, particularly SJS/TEN, during immune therapy, necessitating vigilant monitoring and intervention to prevent adverse outcomes. Early recognition and prompt cessation of treatment are pivotal measures. Future research is warranted to elucidate the pathogenesis of immune-related adverse events and explore more efficacious strategies for prevention and management.

## Data Availability

The original contributions presented in the study are included in the article/supplementary material. Further inquiries can be directed to the corresponding author.
